# Survey of the Effect of Opioid Abuse on the Extent of Coronary Artery Diseases

**DOI:** 10.5539/gjhs.v6n7p83

**Published:** 2014-09-18

**Authors:** B. Rahimi Darabad, J. Vatandust, M. M. Pourmousavi Khoshknab, M. Hajahmadi Poorrafsanjani

**Affiliations:** 1 Reproductive Health Research Center, Urmia University of Medical Science, Urmia, Iran; 2 Urmia University of Medical Sciences-Seyedoshohada Heart Hospital, Urmia, Iran

**Keywords:** opioid, coronary artery disease, risk factors

## Abstract

**Introduction:**

Cardiovascular disease is the most common cause of death in our country. Recently, it has been found that the use of opium like other risk factors can be an independent risk factor for coronary artery disease. Therefore, this study examines the impact of opioid abuse on the extent of coronary artery diseases.

**Methods:**

This study included 1170 individuals, who underwent coronary angiography in Seyed-ol-shohada and Taleghani hospitals, Urmia in 2012, were enrolled. The demographic data included age, sex, medical history, including history of diabetes, hypertension, heart disease and a history of opioid abuse and duration of smoking were extracted and entered in a questionnaire.

**Results:**

The results of this study showed that 121 had taken opium and 1049 patients had not taken the drug. The results of this study showed that coronary artery disease (CAD) in patients with drug use (88.5%) was significantly higher than the group without drug use (72.2 %) (P=0.000). However, significant differences are not exist between the two groups regarding the number of affected coronary arteries (P=0.679).

**Conclusion:**

although risk factors of CAD such as HTN and DM is higher in patients without opium addiction than in addicted patients, but CAD was more created in patients using drugs and this suggests drugs as an important risk factor for coronary artery disease.

## 1. Introduction

Based on available evidence and current studies, the prevalence of opioid abuse nearly tripled over the past twenty years and 2-2/8% of the population in the country according to the latest statistics is included. However, in most of the interior studies the prevalence is much higher among groups of patients (Shirani, Soleymanzadeh, & Esfandbod, 2010). Nowadays it becomes clear that opioids have important effects on many physiologic and pathologic processes that it can undoubtedly have impact on the immune system, respiratory, brain and cardiovascular system undoubtedly noted (Masoomi & Karimzadeh, 2010). On the other hand, morbidity and mortality rates from coronary heart disease are the top cause of mortality and morbidity in Iran (Bjornaas et al., 2008). Physiologically, opioids have affect three types of receptors in cardiac myocytes include, Mu, Kappa, and delta. Studies show that the use of opioids can lead to hypotension, bradycardia, and peripheral vascular relaxation and affect more through potassium channels and calcium exerts (Shirani et al., 2010). In many communities especially in Iran there is a belief based on the protective role of opioids on the cardiovascular system and even in the Middle East opioids sometimes known as lowering blood lipid levels and applying (Shirani et al., 2010; Bjornaas, 2008). So far, internal and external studies on the effects of abuse opioids on the cardiovascular system, have offered conflicting results as some studies raise some risk factors for coronary heart disease such as Hb A1c, coagulation factor 7, fibrinogen and CRP (Bjornaas et al., 2008). and therefore announced that addiction to opioids can increase the risk of mortality from cardiovascular events, and according to some studies, the prevalence of opioid misuse in patients with coronary heart disease is higher than the general population and even after adjusting and considering other risk factors, particularly smoking, abuse of opioids in non-smoking population has been suggested as an independent risk factor for coronary heart disease (Masoomi & Karimzadeh, 2010). Sadat et al. study in Iran indicate increased levels of interleukin 1 in opioid consumers that the inflammatory factor, plays a role in atherosclerosis and thus opioid in this study is discussed as a predisposing factor for coronary heart disease. On the other hand, several studies have provided inverse results such that the chronic use of opioids can result in reducing the severity of coronary heart disease (Masoomi & Karimzadeh, 2010; Saadat et al., 2012). The results of the studies suggest a positive long-term use of opioids in reducing the intensity of coronary heart disease and therefore reducing fatal myocardial infarction, resulting in justification of the role of opioids in reducing the inflammation that associated with atherogenesis and plaque rupture and on the other hand the extended use of opioids, can protect tissue from damage against ischemia and reperfusion and relieve infarction damage (Marmor et al., 2004).

Cardiovascular disease is now a major health priority in all communities, especially in developing countries. The past two decades have changed the geographical distribution of the disease. Therefore, the World Health Organization has placed prevention and treatment of these diseases in health priorities in developing countries, while heart diseases are the fourth factor in mortality in the world and has already caused more deaths in developing countries than developed countries (Vartiainen et al., 1994). In Iran like many other countries the prevalence of risk factors and also non-communicable diseases, which the most abundant of them are heart diseases, are rising. Over 40 percent of deaths in 1991 in Iran were cardiovascular diseases (Sarraf-Zadegan et al., 2003) however, broad programs to control this disease and change behavior patterns and life patterns in Western countries reduced the severity and extent of heart diseases (Puska et al., 1985), and after the widespread implementation of control programs and interventions, the extent of the cardiovascular diseases and mortality has declined about 63 percent (Rose, 1989). However, it is anticipated until 2020 annual heart disease will kill 25 million people in the world. The epidemiological transition due to changes in economic, social and demographic characteristics of the different communities should be identified (Gandelman & Bodenheimer, 2003). Due to this issue, the use of diagnostic, interventional cardiology is increasing day by day. So that, in America between the years 1980-1992 coronary angiography has increased 163% and has reached 15.2 patients in one hundred thousand (Abdullahi et al., 2001). Official estimates of the number of drug abuse and drug users to 2000 reported two million people (3%) (Rahimi et al., 1997). It must be acknowledged that accurate estimates of the prevalence of substance use in different communities include only certain areas of the world that extensive research has been done in this area (Langrod, 1997). Based on the studies more than 15% of the population above 18 years of age in the US have serious problems associated with drug and that two-third is due to alcohol abuse and one-third is due to non-alcohol use (Ahmadi et al., 2003). One of the main factors of addiction is effective factors in the onset of using drugs and undoubtedly stressors including serious physical illness due to the high power of stressing according to the Holmes and Rahe scale play an important role in this field (Abdollahi et al., 2006). In Iran, a major cause of mortality and morbidity is cardiovascular disease and is the leading cause of death in 50% of the population (Hatmi, 2007). The effect of the drug is not fully expressed in different studies, but the study of Sadeghi et al. in 2008 showed that the drug is effective on some risk factors for coronary artery disease (Asgary et al., 2008; Hosseini et al., 2012). According to often contradictory results in relation to the role of opioid abuse in coronary artery disease and the extent and considering the high prevalence of opioid in Iran and on the other hand the relationship with coronary artery disease in Iran, in this study relationship between opioid abuse and the extent of coronary artery disease in patients with symptoms of coronary artery disease that underwent coronary angiography in Sayed-al- Shohada and Taleghani Hospitals in Urmia city are studied. The overall objective of the study is the effect of opioid abuse on the severity of coronary artery disease in patients undergoing coronary angiography.

## 2. Methods

This study was a case - control study that 1170 patients that underwent coronary angiography in Seyed-al-shohada and Taleghani Hospitals in Urmia city in 2012 were enrolled. After approval of the Research Council of Urmia University of Medical Sciences, the project began with the permission of the Research Council. 1170 cases during 2012 in Seyed- al-shohada and Taleghani Hospitals that underwent coronary angiography were enrolled. Patients with the lack of angiographic coronary stenosis were in the control group and those with stenosis separately in one vessel, two-vessel, and three-vessel disease or left main disease formed groups in the study. The demographic data included age, sex, medical history, including history of diabetes, hypertension, heart disease and a history of opioid abuse and duration of smoking were extracted and entered in a questionnaire.

**Exclusion criteria:** Patients who have suffered a myocardial infarction in recent months.

People who do not provide accurate history of drug use the definition of the word drug addict based on the DSM-IV criteria: Someone should have at least three of the following seven items:

1)· Tolerance
2)· Withdrawal symptoms
3)· Taking the drug for more than the recommended amount
4)· Permanent try and failed to reduce the amount of taking drugs
5)· Much time takes to prepare use and recover from the drug
6)· Reduce the social, occupational and recreational activities due to drug use
7)· Continuing to use the drug despite physical and psychological problems (e.g. drug use despite liver disease)

All data were analyzed using statistical software SPSS16 (Dalfard & Nosratian, 2014).

## 3. Findings

**Figure 1:**
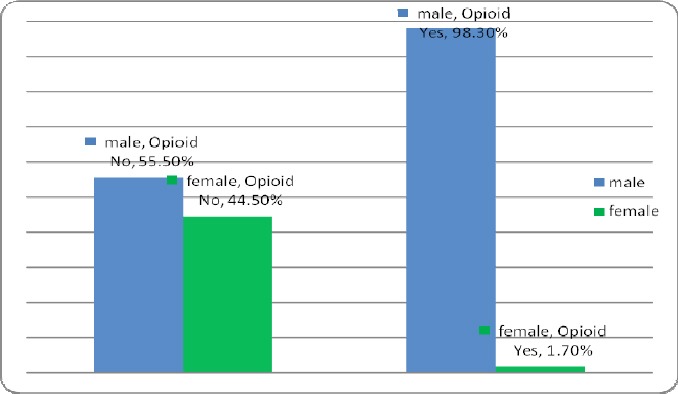
Sex distribution of patients in the group with and without the use of drugs

**Figure 2:**
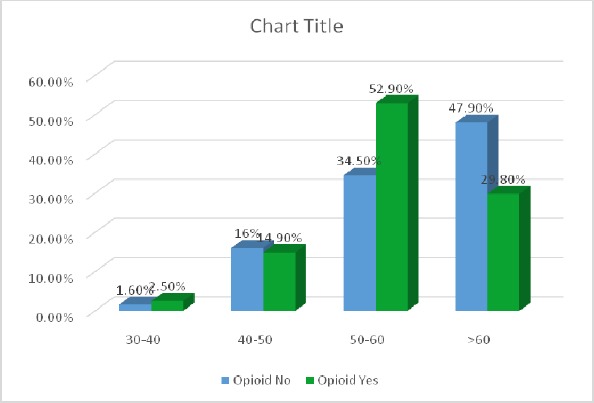
Age distribution of patients in the group with drug use and non-drug use

**Figure 3:**
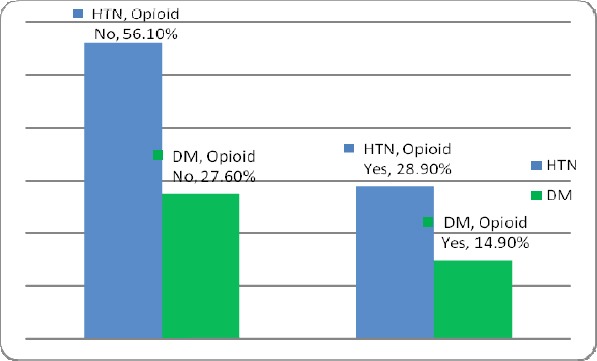
Prevalence of patients with hypertension (HTN) and diabetes mellitus (DM) in patients both with and without using drugs

**Figure 4:**
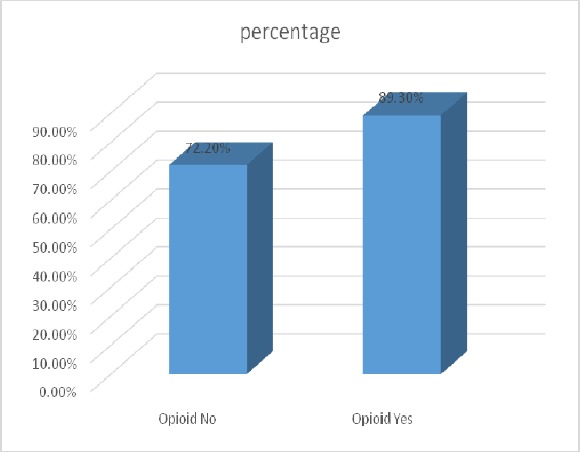
The degree of coronary artery disease (CAD) in both groups with and without the use of drugs

**Figure 5:**
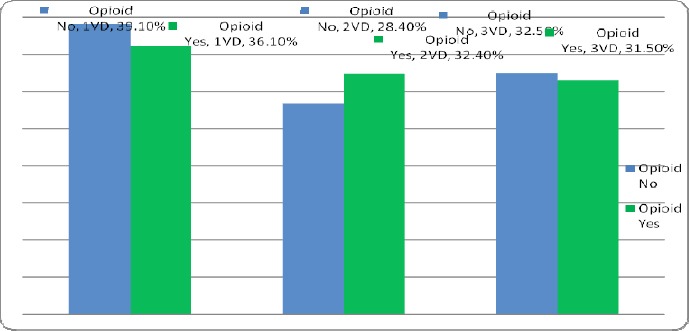
The number of coronary arteries involved in the groups with and without the use of drugs

In this study, 1170 patients who underwent coronary angiography were enrolled. About 121 patients were using drugs, of which 23 patients (19%) orally, 96 patients (79.3%) fumigation and 2 patients (1.7%) with other methods had drug taking. In this group, 2 patients (1.7%) were female and 119 (98.3%) were male. 2.5% (3 patients) in the age group 30-40 years, 14.9% (18 patients) in the age group 40-50 years, 52.9% (64 patients) in the age group 50-60 years and 29.8% (36 patients) were in the age group above 60 years. In this group, 14.9% (18 patients) with diabetes, 28.9 % (35 patients) was diagnosed with hypertension. 99 patients (81.8%) were smoking as well. In this group, 108 patients (89.3%) were with coronary artery disease (CAD). Of these 36.1% had one of the coronary artery diseases (1VD), 32.4% two coronary artery diseases (2VD) and 31.5% three artery diseases (3VD). Also 1049 patients, who underwent coronary angiography, did not use drugs. In this group, 44.5% (467 patients) were female and 55.5% (582 patients) were male (Figure 1). In this group 1.6% (17 patients) was in the age group 30-40 years, 16% (168 patients) in the age group 40-50 years, 34.5% (362 patients) in the age group 50-60 years and 47.9% (502 patients) were in the age group over 60 years (Figure 2). In this group, 27.6% (289 patients) was with diabetes, 56.1% (588 patients) was with hypertension (Figure 3). 310 patients (29.6%) were smoking as well. The people in this group, 757 patients (72.2%) were with coronary artery disease (CAD) (Figure 4). Of these 39.1% (296 patients) had one of the coronary artery diseases (1VD), 28.4% (215 patients) involved two coronary arteries (2VD) and 32.5% (246 patients) involved three arteries (3VD) (Figure 5). The results of this study showed that there were significant differences between the two groups in terms of age (P=0.0001).

The results showed that diabetes is significantly higher in the group without drug use (27.5%) than in the group with drug use (15.6%) (P=0.005). In this study it was found that the group without taking the drug, hypertension (56.1%) was significantly higher than the group taking the drug (28.7%) (P=0.000). Smoking in the group with drug use (82%) was significantly higher than the group without drug use (29.5%) (P=0.000). According to the results of this study it was found that coronary artery disease (CAD) in patients with drug use (89.3%) was significantly higher than the group without drug use (72.2 percent) (P=0.000). However, significant differences were not exist between the two groups regarding the number of affected coronary arteries (P=0.679). Group who were taking opioids, the incidence of CAD in those who had smoked had occurred more, but significant association does not exist between smoking and CAD in this group (P=0.213).

**Table 1:** Comparison of variables between the two groups with and without using drugs

	Group with drug use	Group without drug use	P-Value *
years ≥ 60	8.29 %	9.47 %	001.0
Male	3.98 %	5.55 %	000.0
DM	9.14 %	6.27 %	005.0
HTN	9.28 %	1.56 %	000.0
Smoking	8.81 %	6.29 %	000.0
CAD	3.89 %	2.72 %	000.0

* P Value ≤ 0.05 is significant.

In the group without taking opioid there is a significant correlation between coronary artery disease and smoking and the incidence of CAD in individuals who smoked had occurred more often (P=0.002). According to logistic regression analysis, high age (P=0.000, OR=0.68), sex (P=0.000, OR=0.292), opioid consumption (P=0.019, OR=2.083), diabetes (P=0.000, OR=2.416) and hypertension (P=0.000, OR=2.007) are introduced as a positive risk factor for the incidence of CAD. However, in this study it was observed that in the smoke P=0.069, OR=1.398. In this study there was no need to data because in the group using drugs, although all of the risk factors were lower than the group without taking drugs, but the incidence of CAD was higher in the group taking drugs. The total number of 1170 patients, who underwent coronary angiography, 865 patients had coronary artery disease.

In this group, 11 patients (1.3%) in the age group 30-40 years, 119 patients (13.8%) in the age group 40-50 years, 315 patients (36.4%) 50-60 years and 420 patients (48.6%) were in the age group above 60 years. 34.1% (295 patients) were female and 56.9% (570 patients) were male. 12.5% (108 patients) of the patients was taking drugs that 19 patients (17.6%) orally, 88 patients (81.5%) fumigation and one patient (0.9%) with other methods. In this group, 29.2% (253 patients) were with diabetes and 55.5% (480 patients) were with hypertension.

The patients in this group, 334 patients (38.6%) were smokers. 305 patients had not coronary artery disease. In this group, 9 patients (3%) in the age group 30-40 years, 67 patients (22%) in the age group 40-50 years, 111 patients (36.4%) in the age group 50-60 years and 118 patients (38.7%) in group older than 60 years. 57% (174 patients) in the group was female and 43% (131 patients) were male. 4.6% (13 patients) of the patients in this group were using drugs, of which 4 patients (30.8%) orally, 8 patients (61.5%) fumigation and one patient (7.7%) with other methods. In this group, 17.7% (54 patients) was with diabetes and 46.9% (143 patients) were patients with hypertension. In this group, 75 patients (24.6%) were smokers.

## 4. Discussion

High prevalence of cardiovascular disease is significant worldwide and will include about one-fifth cause of death and is likely to be the most common cause of death worldwide by 2020. Coronary artery disease than any other illness cause death, disability and economic losses in developed countries and is the most common chronic, dangerous and deadly illness in the United States, and more than 12 million people are suffering from ischemic heart disease almost every minute one American die due to coronary artery disease (Demarie et al., 2011). In the population of 70 million in Iran, about 15 million people suffering from cardiovascular diseases. According to the study, cardiovascular disease is the most common cause of death in our country, as about 46% of deaths happen due to it (Sadeghian et al., 2007). So far a number of factors including age, family history, abnormal blood lipids, high blood pressure, diabetes, a history of heart disease are effective in causing disease. International Institute for America’s Health in 2005 stated that each year 23 million people are infected with the disease, 85% of them have at least one risk factor (Sadr Bafghi et al., 2005).

Esteghamati et al. (2006) conducted a study to determine the prevalence of diabetes and other risk factors for heart disease in Tehran and stated that 30% of the units studied had diabetes and 91% had hypertension and 42% of them were smokers (Safaie et al., 2010). Diabetes mellitus is a risk factor for heart disease and accelerated atherosclerosis is often associated with increased risk of angina, myocardial infarction and sudden death. Hypertension also leads to heart disease and studies indicate that there is a strong association between hypertension and ischemic heart disease (Asgary et al., 2008). As well as smoking has a role in the emergence and escalation of the incidence of coronary artery disease and accelerate coronary atherosclerosis in both sexes and all ages, increases the risk of thrombosis, plaque instability and myocardial infarction and death (Truelsen et al., 2003). Recently, it has been found that the use of drugs such as other risk factors can be viewed as an independent risk factor for coronary artery disease and its association with other risk factors increases the risk of ischemic heart disease (Beyranvand et al., 2005). Given that different factors are involved in development of coronary artery disease, but are still not clear which of these factors have a major role in severity and in the development of atherosclerosis (Justin et al., 2002). Drug use is one of the greatest problems of many countries and many people are taking this drug. Opioid that is called drug said to be derived from the opium poppy (Esteghamati et al., 2006). Social and behavioral disorders are not only caused by addiction but also the impact on various aspects of physical health, heavy financial losses on the individual, family and society (Kullo et al., 2004). Since many addictive substances, such as cigarettes, heroin, alcohol, hashish, etc. have been reported in various countries around the world and a large number of researchers are discovering effects of these substances on various aspects of human health. Because cardiovascular diseases among different populations are the first cause of death, it is natural that the bulk of the research has focused on the influence of drugs in patients with cardiovascular diseases (Sposito et al., 2001; Darabian & Abbasi, 2009).

Unfortunately, one of the incentives that exist in the aging population in our society, especially in people who have coronary heart disease or family history of diabetes or are diagnosed with this disease, is a belief in opium effects on blood glucose and blood lipids and reducing cardiovascular diseases and diabetes, Given the high prevalence of cardiovascular diseases in our population the number of drug dependents is increasing day by day (Poormand et al., 2007). Abdullahi et al. determined that age group 28-49 years had the most (52.6%) drug intake (Abdollahi et al., 2006). While the study showed that the highest rates of drug use (52.9%) are in the age group 50-60 years. Hosseini et al. in their study showed that the rate of drug abuse in males patients who had positive angiographic 76.9% and in females with positive angiography 56.1% (Hosseini et al., 2012). However, our results also showed that the rate of drug abuse in patients with positive angiographic was 12.5%. In Masoumi et al. study the rate of drug abuse in patients with CAD was 58.62 (Naderi et al., 2001). However, consumption of opium in Safa et al. study was 16% (Le Moal & Koob, 2007). The most common method of drug abuse in Abdollahi et al. study was inhalation (52.7) (Abdollahi et al., 2006). but in this study people used drugs more as fumigation (79.3%). Our study showed that the prevalence of diabetes in patients taking drugs was 14.9%, and the prevalence of hypertension was 28.9. While, in another study conducted by Masoumi et al. determined that the prevalence of hypertension in people who use drugs and are suffering from mild CAD was 43.21%, and in patients who have severe CAD was 39.55%. The prevalence of diabetes in people who use drugs and are suffering from mild CAD was 22.22%, and in patients who have severe CAD was 28.36 (Ishizaka et al., 2006). In the present study it was found that those taking the drug incidence of CAD were 89.3%, and in those not taking drugs were 72.2. In Masoumi et al. study also determined that 27.09% of those taking the drugs were with mild CAD and 44.84% of patients were with severe CAD (Ishizaka et al., 2006). Sadeghian et al in their study showed that people taking the drug had statistically significant association with coronary artery disease (Sadeghian et al., 2007). Although the results of Marmor et al. study suggest coronary artery disease discounts in long-term use of opioids (Marmor et al., 2004). Sadat et al. study represents the destructive role of drugs in coronary artery disease (Saadat et al., 2012). Our study also showed that significantly coronary artery disease (CAD) in people who use drugs is more than those who did not use drugs (P=0.000). However, in our study we found no significant relationship between the number of arteries involved and drug use (P=0.679). However Sadeghian et al in their study showed that there was a significant association between the number of arteries involved and drug use (P=0.002) (Sarraf-Zadegan, 2003).

## 5. Conclusions

The results of this study showed that although CAD risk factors such as HTN and DM in patients who did not use drugs were more than those who were taking the drug, but CAD was formed more in drug abuse patients and this suggests drugs as an important risk factor for coronary artery disease.

## 6. Suggestions

The results of the present study show that drug use is a risk factor in the incidence of CAD; therefore it is recommended that patients are reduced the disadvantage of taking drugs on the cardiovascular system the use of opioids in patients.
